# A comparison of outcomes of bariatric surgery in patient greater than 70 with 18 month of follow up

**DOI:** 10.1186/s40064-016-3392-x

**Published:** 2016-10-07

**Authors:** Hinali Zaveri, Amit Surve, Daniel Cottam, Christina Summerhays, Austin Cottam, Christina Richards, LeGrand Belnap, Walter Medlin

**Affiliations:** Bariatric Medicine Institute, 1046 East 100 South, Salt Lake City, UT 84102 USA

**Keywords:** Weight loss, Duodenal switch, Elderly, RYGB, LAGB

## Abstract

**Background:**

There is a scarcity of data available to determine the safety and effectiveness of bariatric surgery in the elderly population. Additionally, there are no studies showing the effect of the single anastomosis duodenal switch (SADS) has on the elderly obese, in comparison with other more popular procedures. Here we compare laparoscopic gastric band surgery (LAGB), Laparoscopic Roux-en-Y gastric bypass surgery (LRYGB), and the SADS to analyze the weight loss, perioperative and postoperative morbidity in the patients >70 years of age at a single US center.

**Methods:**

A retrospective analysis was performed on 53 consecutive patients ≥70 years old who underwent weight loss surgery from 2009 to 2015.Weight loss in terms of the percentage excess body mass index lost (%EBMIL), percentage excess weight lost (%EWL) and body mass index (BMI) points lost, resolution of comorbidities, length of stay, early (30-day) and late complication rates were compared using descriptive statistics and non-linear regression analysis.

**Results:**

Of 53 patients, 24 underwent LAGB, 14 underwent LRYGB and 15 underwent SADS. The average patient age was 72.7 ± 2.5 years (range, 70–81.4) and 66 % were females. There was no statistical difference in the demographic data between three groups except for age and sleep apnea. There were no operative or early deaths. There were differences in complication rates between the surgical arms; however, with our small data set statistical significance was not achieved. There was 1 patient who lost to follow up in SADS group. Follow up time period was 18 months.  % EBMIL and BMI reduction showed a statistically significant difference between the procedures, where the SADS had the highest loss of  %EBMIL and BMI points. Comorbidities prevalence decreased post-operatively with SADS having higher percentage of patients who had resolution of their comorbidities.

**Conclusion:**

Each of the three procedures can be performed on patients older than 70 with low morbidity rate. However, when the focus is weight loss alone, the SADS procedure is the most effective of the three procedures in regards to weight loss in the short term for patients older than 70. The SADS is as safe as RYGB but LAGB with all its limitations is still the safest bariatric procedure.

## Background

Life expectancy has been steadily increasing regardless of sex and ethnic background in the USA (Arias 2014). Obesity is known to decrease the quality of life as well as life expectancy (Flegal et al. 2013), and to date only bariatric/metabolic surgeries have achieved significant weight loss, with the corresponding correction or improvement of co-morbidities, improving quality of life.

The rising prevalence of morbid obesity in Unites States has resulted in large population undergoing bariatric surgeries. The elderly population is no exception to this trend. Studies shows that in the range of 60–69 years, 42.5 % women and 38.1 % of men are obese. Among 70–79 years, 31.9 % women and 28.9 % of men are in this condition (Zamboni and Mazzali 2012). Although bariatric surgery in the elderly has been shown to be safe and feasible (Gebhart et al. 2015), there is no current consensus regarding the safety of bariatric surgery in the elderly (Sugerman et al. 2004; Varela et al. 2006; Dorman et al. 2012).

In February 2006, the Centers for Medicare and Medicaid Services (CMS) approved coverage for bariatric surgery as reasonable and necessary for Medicare beneficiaries who meet nationally established criteria for weight loss surgery (Ogden et al. 2007). However, volume of bariatric surgery in the elderly comprise of only 10.1 % of all the bariatric procedures (Gebhart et al. 2015), which is low but has increased significantly from 2.7 % in 1999–2005 (Varela et al. 2006). Although several papers have been published regarding the efficacy of bariatric surgery in older patients (Macgregor and Rand 1993; O’Keefe et al. 2010), the primary concern for these surgeries in elderly is an increased risk for perioperative morbidity and mortality and the adequacy of weight loss due to the relative immobility of the older patients (Printen and Mason 1977).

There are number of bariatric surgeries that are performed on elderly population. However, it is difficult to say which surgery provides better weight loss with less morbidity in these population. Studies in the past have shown that laparoscopic Roux-en-Y gastric bypass (LRYGB) has better weight loss than laparoscopic adjustable gastric banding (LAGB) in elderly population (O’Keefe et al. 2010; Giordano and Victorzon 2015). But there is no published literature about the efficacy of single anastomosis duodenal switch (SADS) in the elderly population. The objective of the present study was to analyze and compare the outcomes in terms of weight loss and perioperative and postoperative morbidity of different bariatric surgeries in patients older than 70 years.

## Methods

Between January 2009 and March 2015, all elderly patients (defined as 70 years and over) undergoing bariatric surgery in our service were identified. They were divided into three groups, each undergoing LAGB, LRYGB and SADS respectively (we did not include sleeve gastrectomy (SG) in our study, as there was only 1 who got this procedure). Patients with revision surgeries were excluded. Data concerning their pre-operative characteristics, peri-operative and post-operative progress was prospectively collated.

Patients were followed up at intervals for a minimum of 18 months. During these visits, patient’s weight and late complication of surgery were recorded. All the patients were given vitamin recommendations by our registered dietician depending on the type of surgery they get. All the patients who underwent LAGB were given multivitamins, vitamin D 3000 IU and calcium as needed. Patients who underwent LRYGB were given multivitamins, vitamin B12-1000 mcg/week, vitamin D 3000 IU and iron 65 mg. Patients who underwent SADS were given ADEK multivitamins, calcium 1800–2400 mg and iron 65 mg.

Descriptive statistics and ANOVA was used to describe and compare the groups preoperatively. Non-linear regression analysis to describe the effect of surgery on the patients at 3, 6, 9, 12, 15, and 18 months. The Chi square test was used when calculating statistical significance among the comorbidities, as well as the gender ratio. All the statistical analysis was performed through SigmaPlot software.

## Surgical technique

Patients were selected for each surgery based on when they came in and the surgeon they chose. All the surgeries were performed by two surgeons at the same practice. Our method of band placement has been described in detail previously (Cottam et al. 2006).

The surgical technique used for LRYGB has been published previously (Cottam et al. 2009; Fisher et al. 2007). Briefly, we create a 5 cm pouch using a linear stapler and attach a 150 cm Roux limb using a 25 mm EEA technique and an Orvil device (Medtronic). Our biliopancreatic limb is 30 cm long.

The surgeons in our practice began informing patients of the SADS option in 2013. Patients chose SADS based on an extensive preoperative educational experience and signed a specific informed consent detailing the SADS procedure that included a diagram of the proposed operation. The surgical technique for SADS has slight modification from the loop duodenal switch (LDS) as published by Torres and Huang (Sánchez-Pernaute et al. 2010; Sánchez-Pernaute et al. 2015; Sánchez-Pernaute et al. 2013; Sanchez-Pernaute et al. 2015; Huang et al. 2014). Briefly, although the anastomosis is the same our common channel is 300 cm rather than 250 cm. The SG is over a 40 french bougie rather than a 56. This makes a smaller SG for greater restriction and a longer common channel for less chance of malabsorption. Torres had a 1 % malnutrition rate at 250 cm, we hypothesized that it would be near zero at 300 cm (Sanchez-Pernaute et al. 2015). Suture lines on the LRYGB and SADS.

## Results

### Demographics

Fifty-three patients aged 70 or over underwent bariatric surgery during the study period. The average patient age was 72.7 ± 2.5 years (range, 70–81.4). Of 53 patients, 24 underwent LAGB, 14 underwent LRYGB and 15 underwent SADS. The patient demographic and baseline information are seen in Table 1. The majority of patients were females (66 %), with no statistically significant difference (p = .8) in female preponderance between the three groups. All patients had at least one obesity-related comorbidity. As shown there was no statistical difference in profiles between three groups except for age and sleep apnea.

**Table 1:** Patients baseline demographic data

	LAGB	LRYGB	SADS	p value
N	24	14	15	
Male/female	6/18	5/9	7/8	.38
Age^a^	71.9 ± 2	73.1 ± 2.5	73.6 ± 2.9	.08
Weight^a^	265.5 ± 35	253.2 ± 50.2	273.8 ± 36.3	.46
BMI^a^	44.3 ± 5.9	40.8 ± 5.2	44.2 ± 5.4	.17
*Comorbidity*				
Sleep apnea	10/24 (42 %)	10/14 (71 %)	11/15 (73 %)	.08
Diabetes	12/24 (50 %)	6/14 (42 %)	7/15 (47 %)	.97
GERD	6/24 (25 %)	6/14 (43 %)	8/15 (53 %)	.19
Hypertension	19/24 (79 %)	12/14 (86 %)	11/15 (73 %)	.71

*LAGB* laparoscopic gastric banding, *LRYGB* laparoscopic Roux-en-Y gastric bypass, *SADS* single anastomosis duodenal switch, *GERD* gastroesophageal reflux disease

^a^Values are expressed as mean ± standard deviation

### Perioperative outcomes

All surgeries were performed laparoscopically with no conversion to open. Mean hospital stay was 1.4 ± .9 days for LAGB (range, 1–5), 3.1 ± 4.1 days for LRYGB (range, 1–17) and 2.1 ± 1.6 days for SADS (range, 1–7).

The complications for LAGB, LRYGB and SADS are summarized in Table 2 respectively. Statistical comparisons are unable to be performed since each complication was unique. There was no re-admission within 30 days of discharge after LAGB, 1 after LRYGB and 2 after SADS, with no statistical significant difference in re-admission rates between the three procedures. There was no 30-day mortality in any patients undergoing any one of three procedures. Highest complication rates were seen after LAGB, though more serious complications were seen after LRYGB. Only 1 patient who underwent LRYGB needed revision of gastrojejunal (GJ) anastomosis. This patient had stricture at GJ anastomosis that needed dilation, however, 1 year later, the same patient developed ulcers and fistula and needed revision. Similarly, 1 patient after SADS also needed common channel lengthening because of chronic diarrhea.

**Table 2:** Complications seen with all the three procedures

LAGB	LRYGB	SADS
Early	Early	Early
Pneumonia-1	Pneumonia-1	Acute cholecystitis, sub hepatic abcess-1^i^
Reflux-1	Wound infection-1	Wound infection-1
	Stricture-1^e^	Gastro cutaneous fistula-1^j^
	Leak and abcess-1^f^	
Total early complication rate-8.3 %	Total early complication rate-28.5 %	Total early complication rate-20 %
Late	Late	Late
Nausea and vomiting-5^a^	Stricture-1^g^	Diarrhea-2^k^
Reflux-3^b^	Reflux-2^h^	Stricture needing dilation-1
Weight regain-4	Chronic diarrhea-1	
Slipped lap band-2^c^		
Erosion of lap band port-1^d^		
Total late complication rate-62.5 %	Total late complication rate-28.5 %	Total late complication rate-20 %

*LAGB* laparoscopic gastric banding, *LRYGB* laparoscopic Roux-en-Y gastric bypass, *SADS* single anastomosis duodenal switch

*In LAGB group*

^a^All the 5 patient had nausea and vomiting because of band being too tight. All of them got their band adjusted

^b^One of the patient had severe reflux, couldn’t keep any food down and, needed a larger band replacement

^c^One of the patient with slipped band had inverted port 2 years later and needed the band removal

^d^Patient with the erosion of lap band port needed the surgery for the exchange of lap band port for low profile port

*In LRYGB group*

^e^One patient complained of GERD and underwent EGD. EGD showed stricture at the gastric outlet obstruction requiring dilation

^f^This patient had history of multiple abdominal surgeries who underwent elective LRYGB, left inguinal hernia repair and hiatal hernia repair. Intraoperatively, patient had bladder perforation requiring bladder reconstruction. On post-operative day 1, patient developed intra-abdominal leak with bile noted on JP drain. Patient was taken back to the operating room and underwent exploratory laparotomy with incision and drainage, removal of abdominal mesh, and partial omentectomy due to marginally vascularized omentum. Patient was transferred to intensive care unit (ICU) and was extubated. On post-operative day 10, patient developed sepsis and abdominal abscess. Patient was treated with antibiotics and was placed on total parental nutrition (TPN) because of severe malnutrition. At last, patient developed acute renal insufficiency secondary to pre-renal azotemia. Patient got discharged after 17 days and was transferred to rehab for recovery

^g^This patient complained of progressive dysphagia, who also underwent EGD which showed Gastrojejunal (GJ) stricture needing dilation. This patient after 1 year developed ulcers and fistula and underwent revision of GJ anastomosis

^h^One of the 2 patients who complained of reflux also had severe abdominal pain. EGD showed hiatal hernia and bifid gastric pouch. Patient underwent hiatal hernia repair with mesh and partial gastrectomy

*In SADS group*

^i^This patient had acute cholecystitis and sub hepatic abscess needing re-admission within 30 days of discharge. The patient underwent cholecystectomy and drainage of abscess

^j^This is the second patient who needed re-admission within 30 days of discharge for the treatment of gastro cutaneous fistula

^k^One of the patients had chronic diarrhea, excessive weight loss, and hypoalbuminemia requiring common channel lengthening approximately 1 year after SADS

### Weight loss and resolution of comorbidities

**Fig. 1:**
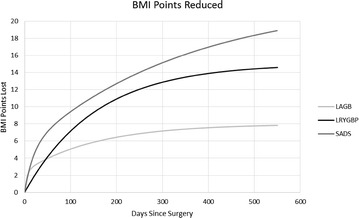
BMI points lost between three procedures during 18 months. *LAGB* laparoscopic gastric banding, *LRYGB* laparoscopic Roux-en-Y gastric bypass, *SADS* single anastomosis duodenal switch

Weight loss results between the procedures were statistically significantly different for percentage excess body mass index lost (%EBMIL) and Body mass index (BMI) points lost for most of the variable studied (Table 3; Fig. 1).  %EBMIL at 12 months was 36.6, 80.6 and 85.5 and at 18 months was 372, 88.4 and 100.6 for LAGB, LRYGB and SADS respectively. Similarly, BMI points lost at 12 months was 7.5, 13.6 and 16.4 and at 18 months was 7.8, 14.6 and 18.9 for LAGB, LRYGB and SADS respectively. However, there was no statistical difference between percentage excess weight losses (%EWL) between three groups for all the variables (Table 4). SADS lost highest amount of weight at all the given time points, while LAGB lost least amount of weight at all the given time points. There was 1 patient in SADS which completely lost to follow-up.

**Table 3:** Percentage excess BMI lost at 3, 6, 9, 12, 15, and 18 months for the LAGB, LRYGB and SADS

%EBMIL	3 months	6 months	9 months	12 months	15 months	18 months
LAGB	25.6	32.4	35.3	36.6	37	37.2
CI	(22.1, 29.1)	(29.4, 35.5)	(32.7, 38)	(33.3, 39.8)	(33.2, 40.8)	(33.1, 41.4)
N	23/24 (95.8 %)	22/24 (91.7 %)	21/24 (87.5 %)	21/24 (87.5 %)	21/24 (87.5 %)	20/24 (83.3 %)
LRYGB	41	60.2	72.5	80.6	85.2	88.4
CI	(37.8, 45.5)	(55.3, 65.2)	(68, 77)	(76.5, 84.8)	(80.2, 90.1)	(82.1, 94.7)
N	12/14 (85.7 %)	12/14 (85.7 %)	12/14 (85.7 %)	12/14 (85.7 %)	12/14 (85.7 %)	11/14 (78.6 %)
SADS	49.1	63.4	75.2	85.5	94.1	100.6
CI	(41.8, 56.5)	(57.6, 69.2)	(69.8, 80.7)	(79.6, 91.4)	(88, 100.2)	(94, 107.3)
N	14/15 (93.3 %)	12/15 (80 %)	10/15 (66.7 %)	9/11 (81.8 %)	7/10 (70 %)	7/8 (87.5 %)
p value	>.05	>.05	<.05	<.05	>.05	<.05

*LAGB* laparoscopic gastric banding, *LRYGB* laparoscopic Roux-en-Y gastric bypass, *SADS* single anastomosis duodenal switch,  *%EBMIL* percentage excess BMI point lost, *CI* confidence interval, *N* number of patients/percentage follow-ups available

**Table 4:** Percentage excess weight loss (%EWL) at 3, 6, 9, 12, 15, and 18 months, for the LAGB, LRYGB, and the SADS

%EWL	3 months	6 months	9 months	12 months	15 months	18 months
LAGB	22.5	28.4	29.9	30.3	30.4	30.4
CI	(19.3, 25.7)	(26.2, 30.5)	(27.4, 32.3)	(27.5, 33)	(27.5, 33.2)	(27.5, 33.3)
N	23/24 (95.8 %)	22/24 (91.7 %)	21/24 (87.5 %)	21/24 (87.5 %)	21/24 (87.5 %)	20/24 (83.3 %)
LRYGB	35.2	53.5	63.6	69.1	71.7	73.2
CI	(31.5, 38.9)	(49.9, 57.1)	(60.3, 66.9)	(65.3, 72.9)	(67.2, 76.1)	(68, 78.3)
N	12/14 (85.7 %)	12/14 (85.7 %)	12/14 (85.7 %)	12/14 (85.7 %)	12/14 (85.7 %)	11/14 (78.6 %)
SADS	40.4	50.6	59.3	67.4	74.2	80.3
CI	(34.5, 46.3)	(45.7, 55.5)	(55, 63.7)	(62.6, 72.2)	(69.1, 79.4)	(74.7, 86.2)
N	14/15 (93.3 %)	12/15 (80 %)	10/15 (66.7 %)	9/11 (81.8 %)	7/10 (70 %)	7/8 (87.5 %)
p value	>.05	>.05	>.05	>.05	>.05	>.05

*LAGB* laparoscopic gastric banding, *LRYGB* laparoscopic Roux-en-Y gastric bypass, *SADS* single anastomosis duodenal switch,  *%EWL* percentage excess weight loss, *CI* confidence interval, *N* number of patients/percentage follow-ups available at each time point

As shown in Table 5, all metabolic comorbidities decreased in prevalence post-operatively and this was comparable between the three groups. For simplicity, absolute resolution of comorbidities is given here, as defined by no need for medications. As shown, SADS had the highest resolution of comorbidities compared to other two groups, with 100 % resolution of diabetes.

**Table 5:** Resolution of comorbidities

	LAGB (n = 24)	LRYGB (n = 14)	SADS (n = 15)	p value
Sleep apnea	5/10 (50 %)	6/10 (60 %)	5/10 (50 %)	.96
Diabetes	8/12 (66.7 %)	5/6 (83.3 %)	7/7 (100 %)	.84
GERD	3/6 (50 %)	3/6 (50 %)	4/7 (57.1 %)	.98
Hypertension	15/19 (78.9 %)	6/12 (50 %)	10/11 (90.9 %)	.64

*LAGB* laparoscopic gastric banding, *LRYGB* laparoscopic Roux-en-Y gastric bypass, *SADS* single anastomosis duodenal switch, *GERD* gastroesophageal reflux disease

## Discussion

The prevalence of patients that are both obese and elderly is increasing, and is likely continue to do so (Sturm 2003; Fakhouri et al. 2012). Our study is first of its kind which demonstrates three different procedures in the elderly in a single practice with the same pre and post-operative education regime. It clearly shows the medium-term efficacy of bariatric surgery in the obese elderly patients: good percentage excess weight loss and reduction in comorbidities following bariatric surgery.

Our study demonstrates SADS had fewest number of peri-operative complications when compared to other two. These results are very comparable to the younger population undergoing SADS at our institution only (Brian et al. 2016). However, these fewer complications never became statistically significant. So we can’t point to a single operation as being the safest in the geriatric population but we can say that any operation performed for obesity should have complication rates similar to non-geriatric patient populations (Zamboni and Mazzali 2012; Giordano and Victorzon 2015; Nassif et al. 2015; Fatima et al. 2006; Trieu et al. 2007). This should allow clinicians to make operational choices based on patient characteristics other than age.

There is a detrimental impact of age on wound healing in all tissues. Aging intrinsically and extrinsically impacts the skin, leading to atrophy, progressive loss of function, increased vulnerability to the environment, and decreased homeostatic capability (Giordano and Victorzon 2015). Diminished extracellular matrix slows wound healing. These factors together with higher comorbidity prevalence in elderly patients may explain the higher complication rates. Even despite this high peri-operative complications, in the elderly obese population, bariatric surgery significantly improves overall 5 and 10-year survival (Arteburn et al. 2015).

There are already several studies that states the fact that the LRYGB surgery is more effective than the LAGB surgery, both in younger and in older population. (Arterburn et al. 2014; Colquitt et al. 2014; Chapman et al. 2004; Daigle et al. 2016; Zuegel et al. 2012; Angrisani et al. 2013). SADS is at least as effective as the LAGB if not more. So the fact that the weight loss for SADS is better than the LAGB is not surprising. What is of interest, in this case, is the comparison between the LRYGB and the SADS in terms of weight loss. Our previously published literature, have clearly demonstrated weight loss better with SADS when compared to LRYGB in younger population, and this study reciprocates our results with the elderly population (Cottam et al. 2015). The percentage excess BMI lost from the SADS differs statistically from the LRYGB from 9 to 18 months as shown in Fig. 1 while Table 3 also speaks in favor of the SADS over the LRYGB while considering BMI reduction. This is similar to what Torres reported in his paper (Sánchez-Pernaute et al. 2010).

Another significant factor in this paper is the 18-month time period that we chose to follow these patients. We chose this amount as it allowed us to capture the peak of each operative procedures weight loss. SADS peaks at 18 months while the LAGB peaks at 12 months and stays flat. The LRYGB loses weight rapidly to 15 months and then almost immediately begins to gain weight. These weight loss characteristics mirror those seen in non-geriatric patients (Cottam et al. 2006; Sánchez-Pernaute et al. 2010).

Bariatric surgery has been shown to be effective for obesity comorbidity problems such as sleep apnea (Sugerman et al. 1992; Charuzi et al. 1992), GERD (Smith et al. 1997), diabetes mellitus (DM) (Pories et al. 1992; Pories et al. 1995) and hypertension (Foley et al. 1992; Carson et al. 1994). We have noted that SADS was associated with 100 % resolution of diabetes mellitus compared to 85.7 % after LRYGB. The SADS group had a higher percentage of DM patients. The higher percentage of DM in the SADS we thought it would have made the SADS patients more metabolically challenged at 18 months and thus lose less weight than the LRYGB patients. The literature is mixed on whether DM affects weight loss (Cottam et al. 2009; Brian et al. 2016; Wise et al. 2016; Cazzo et al. 2014; Roslin et al. 2015). The SADS patients in our study were able to lose as much or even more weight as the LRYGB even with more diabetic patients.

Several studies have suggested that LRYGB is an effective surgery for GERD in morbidly obese patients (Braghetto et al. 2012; Varela et al. 2009). All the three group had higher rates of GERD pre-op and remarkably low rates of GERD post-op with SADS having the lowest prevalence post-operatively. This may reflect our aggressive nature in the diagnosis hiatal hernia preoperatively and its treatment intra-operatively. We must acknowledge that this study only looked at acid reducing medication use pre and post operatively and not Ph studies or GERD scores. With 8 patients taking anti-acid medications pre-operatively in SADS group and only 3 taking at 18 months compared to 6 taking pre-operatively in LRYGB group and 3 taking at 18 months does demonstrates the superior nature of SADS treating GERD as compared to both the other groups. This is very much supported by the study done at our institution on the effect of SADS to treat GERD in morbidly obese patients (Zaveri et al. 2015).

Age alone should not be an absolute contradiction for bariatric surgery. Indications should be carefully evaluated in the light of routine pre-operative tests and discussed with the patients knowing that there are some risks, and that the results might not be as good as they might expect.

The current study has several limitations and potential bias influencing these findings. The number of patients in our series is small, and there is a need for much larger studies to confirm our findings. Another limitation of this study is that all the three groups were not matched in terms of pre-operative demographics; however, there were few significant differences between the groups with respect to age and sleep apnea.

We used self-reported final weights in the many cases, which may be prone to unreliability. However, our study used non-linear regressions to make the best possible comparison between the three groups’ weight loss. The use of non-linear regression allowed our line of best fit to have a higher correlation coefficient when compared with a linear regression. Regression analysis allowed us to include all patients’ data and have the highest accuracy possible.

Another interesting, but we feel non-significant, limitation is the fact that 2 surgeons performed the procedures in the study. Each surgeon feels strongly about the benefits of all the procedures that they offer. Each surgeon may offer LAGB, LRYGB or SADS to slightly different patients. Additionally, this paper is not meant to be the last word in comparing the three different procedure in elderly obese population. Our short term follow up is another limitation of this study. As such many more papers and long term results will be needed to reaffirm the finding of this study.

The risk of diarrhea and malabsorption is already high in elderly population compared to younger population (Holt 2001), most surgeons fear to choose biliopancreatic diversion with duodenal switch (BPD/DS) over LRYGB with the fear of more malnutrition. Since this was a retrospective study, it was not meant to look into malabsorption rate. While this would have strengthened the comparisons between the three procedures, this was another limitation of the study. However, the SADS with its 300 cm common channel empirically should be expected to have less malnutrition and malabsorption than the BPD/DS with its shorter common channel and Roux limbs. The way to show increase malabsorption rate is to see the percentage of patients complaining of diarrhea or oily stools. In LRYGB, there was 1 patient who complained of chronic diarrhea, in spite treating with various antibiotics. In SADS, there were 2 patients who complained of diarrhea, one was treated empirically, while another needed roux limb lengthening to 450 cm. Both the patients were symptom free at the following visits.

The other weakness of this study is to obtain 100 % follow-ups. This is a common problem in United States, where insurance pre-authorization is required, and the cost of laboratory studies are at least partially borne by patients. However, all the efforts were made to maintain their care. They were seen at each visit by a registered dietician who offered behavioral modification suggestion if they were regaining weight.

We also didn’t collect the quality of life questionnaire, which is the other limitation of our study. Despite these limitations, this is the first study to demonstrate the effect of SADS on the elderly obese population (>70 years of age) and compare the outcomes with LRYGB and LAGB from a single practice. Potential avenues for further research would include analyzing the relative efficacy of these bariatric surgeries in patients older than 70 years of age as compared with obese younger population, given that the former may have a greater burden of acquired obesity-related comorbidities.

## Conclusion

In patients older than 70 years of age, bariatric procedures represent effective treatment option until better medical management becomes available to them. Age should not be considered as an absolute impediment for any kind of bariatric procedures. As every patient is different, one must always take into account the patient history when deciding which procedure will be the most effective. The LAGB, despite having the least weight loss, is still vastly superior to medical weight loss. The SADS patient experienced the most effective weight loss with an average of 80 % EWL at 18 months, as well as high percentage of resolution of their comorbidities, this is not surprising since it does have the greatest amounts of fat malabsorption. The SADS is as safe as RYGB but LAGB with all its limitations is still the safest bariatric procedure.

## Abbreviations

SADS: single anastomosis duodenal switch
LAGB: laparoscopic gastric band surgery
LRYGB: laparoscopic Roux-en-Y gastric bypass surgery
EBMIL: excess body mass index lost
EWL: excess weight lost
BMI: body mass index
SG: sleeve gastrectomy
LDS: loop duodenal switch
IRB: Institutional Review Board
GERD: gastroesophageal reflux disease
DM: diabetes mellitus
BPD/DS: biliopancreatic diversion with duodenal switch

## References

[CR1] Angrisani L, Cutolo PP, Formisano G, Nosso G, Vitolo G (2013). Laparoscopic adjustable gastric banding versus Roux-en-Y gastric bypass: 10-year results of a prospective, randomized trial. Surg Obes Relat Dis..

[CR2] Arias E (2014). United States life tables, 2010. Natl Vital stat Rep..

[CR3] Arteburn DE, Olsen MK, Smith VA (2015). Association between bariatric surgery and long term survival. JAMA.

[CR4] Arterburn D, Powers JD, Toh S (2014). Comparative effectiveness of laparoscopic adjustable gastric banding vs laparoscopic gastric bypass. JAMA Surg..

[CR5] Braghetto I, Korn O, Csendes A, Gutierrez L, Valladares H, Chacon M (2012). Laparoscopic treatment of obese patients with gastrooesophageal reflux disease and Barrett’s esophagus: a prospective study. Obes Surg.

[CR6] Brian M, Daniel C, Richie G, Samuel C, Hinali Z, Amit S, Mitchell SR (2016) Stomach intestinal pylorus sparing surgery for morbid obesity: retrospective analysis of our preliminary experiences. Obes Surg 10.1007/s11695-016-2077-4 26932811

[CR7] Carson JL, Ruddy ME, Duff AE (1994). The effect of gastric bypass surgery on hypertension in morbidly obese patients. Ann Intern Med.

[CR8] Cazzo E, da Silva FP, Pareja JC, Chaim EA (2014). Predictors for weight loss failure following Roux-en-Y gastric bypass. Arq Gastroenterol.

[CR9] Chapman AE, Kiroff G, Game P (2004). Laparoscopic adjustable gastric banding in the treatment of obesity: a systematic literary review. Surgery..

[CR10] Charuzi I, Lavie P, Peiser J (1992). Bariatric surgery in morbidly obese sleep-apnea patients: short- and long-term follow-up. Am J Clin Nutr.

[CR11] Colquitt JL, Pickett K, Loveman E, Frampton GK. Surgery for weight loss in adults. Cochrane Database Syst Rev. 2014 8 10.1002/14651858.CD003641.pub4 PMC9028049 25105982

[CR12] Cottam DR, Atkinson J, Anderson A, Grace B, Fisher B (2006). A case-controlled matched-pair cohort study of laparoscopic Roux-en-Y gastric bypass and lap-band patients in a single US center with three-year follow-up. Obes Surg.

[CR13] Cottam DR, Fisher B, Sridhar V, Atkinson J, Dallal R (2009). The effect of stoma size on weight loss after laparoscopic gastric bypass surgery: results of a blinded randomized controlled trial. Obes Surg.

[CR14] Cottam A, Cottam D, Medlin W, et al (2015) A matched cohort analysis of single anastomosis loop duodenal switch versus Roux-en-Y gastric bypass with 18-month follow-up. Surg Endosc 10.1007/s00464-015-4707-7 26694182

[CR15] Daigle C, Andalib A, Corcelles R, Cetin D, Schauer P, Brethauer S (2016). Bariatric and metabolic outcomes in the super-obese elderly. Surg Obes Relat Dis..

[CR16] Dorman RB, Abraham AA, Al-Refaie WB, Parsons HM, Ikramuddin S, Habermann EB (2012). Bariatric surgery outcomes in the elderly: an ACS NSQIP study. J Gastrointest Surg..

[CR17] Fakhouri TH, Ogden CL, Carroll MD (2012). Prevalence of obesity among older adults in the United States, 2007–2010. NCHS Data Brief..

[CR18] Fatima J, Houghton SG, Iqbal CW (2006). Bariatric surgery at the extremes of age. J Gastrointest Surg..

[CR19] Fisher BL, Atkinson JD, Cottam D (2007). Incidence of gastroenterostomy stenosis in laparoscopic Roux-en-Y gastric bypass using 21- or 25-mm circular stapler: a randomized prospective blinded study. Surg Obes Relat Dis..

[CR20] Flegal KM, Kit BK, Orpana H, Graubarb BI (2013). Association of all-cause mortality with overweight and obesity using standard body mass index categories: a systemic review and meta-analysis. JAMA.

[CR21] Foley EF, Benotti PN, Borlase BC (1992). Impact of gastric restrictive surgery on hypertension in the morbidly obese. Am J Surg.

[CR22] Gebhart A, Young MT, Nguyen NT (2015). Bariatric surgery in the elderly: 2009–2013. Surg Obes Relat Dis..

[CR23] Giordano S, Victorzon M (2015). Bariatric surgery in elderly patients: a systemic review. Clinical inter in aging..

[CR24] Holt PR (2001). Diarrhea and malabsorption in the elderly. Gastroenterol Clin North Am.

[CR25] Huang CK, Ahluwalia JS, Garg A, et al (2014). Novel metabolic/bariatric surgery—loop duodenojejunal bypass with sleeve gastrectomy (LDJB-SG), essentials and controversies in bariatric surgery, InTech, Rijeka. doi: 10.5772/58890. http://www.intechopen.com/books/essentials-and-controversies-in-bariatric-surgery/novel-metabolic-bariatric-surgery-loop-duodenojejunal-bypass-with-sleeve-gastrectomy-ldjb-sg

[CR26] Macgregor AM, Rand CS (1993). Gastric surgery in morbid obesity: outcomes in patient aged 55 years and over. Arch Surg.

[CR27] Nassif PA, Malafaia O, Ribas-Filho JM, Czeczko NG, Garcia RF, Ariede BL (2015). When and why operate elderly obese. Arg Bras Cir Dig..

[CR28] O’Keefe KL, Kemmeter PR, Kemmeter KD (2010). Bariatric surgery outcomes in patients aged 65 years and older at an American Society for Metabolic and Bariatric Surgery Center of Excellence. Obes Surg.

[CR29] Ogden CL, Carrol MD, McDowell MA, Flegal KM (2007) Obesity among adults in the United States-no change since 2003–2004, NCHS data brief no. 1, Hyatsville, MD, national Center for Health statistics 19389313

[CR30] Pories WJ, MacDonald KG, Morgan EJ (1992). Surgical treatment of obesity and its effect on diabetes: 10 years follow up. Am J Clin Nutr.

[CR31] Pories WJ, Swanson MS, MacDonald KG (1995). Who would have thought it? An operation proves to be the most effective therapy for adult-onset diabetes mellitus. Ann Surg.

[CR32] Printen KJ, Mason EE (1977). Gastric bypass for morbid obesity in patients more than fifty years of age. Surg Gynecol Obstet..

[CR33] Roslin MS, Gagner M, Goriparthi R, Mitzman M (2015). The rationale for a duodenal switch as the primary surgical treatment of advanced type 2 diabetes mellitus and metabolic disease. Surg Obes Relat Dis..

[CR34] Sanchez-Pernaute A, Rubio MA, Cabrerizo L, Ramos-Levi A, Perez Aquirre E, Torres A (2015). Single-anastomosis duodenoileal bypass with sleeve gastrectomy (SADI-S) for obese diabetic patients. Surg Obes Relat Dis..

[CR35] Sánchez-Pernaute A, Herrera MA, Pérez-Aguirre ME (2010). Single anastomosis duodeno-ileal bypass with sleeve gastrectomy (SADI-S) one to three-year follow-up. Obes Surg..

[CR36] Sánchez-Pernaute A, Rubio MÁ, Pérez Aguirre E, Barabash A, Cabrerizo L, Torres A (2013). Single-anastomosis duodenoileal bypass with sleeve gastrectomy: metabolic improvement and weight loss in first 100 patients. Surg Obes Relat Dis..

[CR37] Sánchez-Pernaute A, Rubio MÁ, Conde M, Arrue E, Pérez-Aguirre E, Torres A (2015). Single-anastomosis duodenoileal bypass as a second step after sleeve gastrectomy. Surg Obes Relat Dis..

[CR38] Smith SC, Edwards CB, Goodman GN (1997). Symptomatic and clinical improvement in morbidly obese patients with gastroesophageal reflux disease following Roux-en-Y gastric bypass. Obes Surg.

[CR39] Sturm R (2003). Increase in clinically severe obesity in the United States, 1986–2000. Arch Intern Med..

[CR40] Sugerman HJ, Fairman RP, Sood RK (1992). Long-term effects of gastric surgery for treating respiratory insufficiency of obesity. Am J Clin Nutr.

[CR41] Sugerman HJ, DeMaria EJ, Kellum JM, Sugerman EL, Meador JG, Wolfe LG (2004). Effects of bariatric surgery in older patients. Ann Surg.

[CR42] Trieu HT, Gonzalvo JP, Szomstein S (2007). Safety and outcomes of laparoscopic gastric bypass surgery in patients 60 years of age and older. Surg Obes Relat Dis..

[CR43] Varela JE, Wilson SE, Nguyen NT (2006). Outcomes of bariatric surgery in the elderly. Am Surg.

[CR44] Varela JE, Hinojosa MW, Nguyen NT (2009). Laparoscopic fundoplication compared with laparoscopic gastric bypass in morbidly obese patients with gastrooesophageal reflux disease. Surg Obes Related Dis..

[CR45] Wise ES, Hocking KM, Kavic SM (2016). Prediction of excess weight loss after laparoscopic Roux-en-Y gastric bypass: data from an artificial neural network. Surg Endosc.

[CR46] Zamboni M, Mazzali G (2012). Obesity in the elderly: an emerging health issue. Int J Obes (Lond)..

[CR47] Zaveri H, Surve A, Cottam D (2015). Stomach intestinal pylorus sparing surgery (SIPS) with laparoscopic fundoplication (LF): a new approach to gastroesophageal reflux disease (GERD) in the setting of morbid obesity. SpringerPlus..

[CR48] Zuegel NP, Lang RA, Huttl TP (2012). Complications and outcomes after laparoscopic bariatric surgery: LAGB versus LRYGB. Langenbecks Arch Surg..

